# 2bRAD-M Reveals the Characteristics of Urinary Microbiota in Overweight Patients with Urinary Tract Stones

**DOI:** 10.3390/biomedicines13051197

**Published:** 2025-05-14

**Authors:** Pengfei Wu, Jingcheng Zhang, Wentao Zhang, Fuhang Yang, Yang Yu, Yuke Zhang, Guangchun Wang, Haimin Zhang, Yunfei Xu, Xudong Yao

**Affiliations:** 1 Department of Urology, Shanghai Tenth People’s Hospital, Clinical Medical College of Nanjing Medical University, Shanghai 200072, China; 2 Department of Urology, Shanghai Tenth People’s Hospital, School of Medicine, Tongji University, Shanghai 200072, China; 3 Urologic Cancer Institute, School of Medicine, Tongji University, Shanghai 200072, China

**Keywords:** obesity, urinary tract stone (UTS), microbiota, urine, 2bRAD-M

## Abstract

**Background:** Urinary tract stone (UTS) is a common disease significantly impacting human health. Obesity influences stone formation and increases UTS incidence, yet the differences in the urinary microbiota and pathways between overweight and healthy-weight UTS patients remain unclear. **Methods:** In this study, 16 patients were analyzed: 8 overweight and 8 healthy-weight UTS patients. Bladder urine samples were collected during surgery, and DNA was extracted for microbial analysis using 2bRAD markers. Microbial diversity and KEGG pathway differences were studied. **Results:** The results showed that overweight UTS patients had a significantly higher urinary microbial diversity than healthy-weight patients. The analysis identified differences in microbiota at various taxonomic levels. LEfSe analysis revealed *Sphingomonas_paucimobilis* as abundant in overweight patients, while *Bifidobacterium_piotii* dominated in healthy-weight patients. Key species, including *Ralstonia_sp000620465*, *Sphingomonas_paucimobilis*, and *Campylobacter_D_coli*, were identified. KEGG analysis highlighted enriched pathways in overweight UTS patients, including the porphyrin and chlorophyll metabolism, fatty acid metabolism, amino acid degradation, and renin–angiotensin and mineral absorption pathways. **Conclusions:** This study is the first to use 2bRAD-M microbiome analysis to compare the urinary microbiota between overweight and healthy-weight UTS patients. It identified significant microbiota and pathway differences, suggesting a link between microbiota imbalance, obesity, and stone formation. These findings provide potential targets for further research on obesity-related stone susceptibility mechanisms.

## 1. Introduction

Urinary tract stone (UTS) is a common and frequently occurring disease that seriously affects human health, accounting for the majority of urology inpatients, and its incidence rate remains high for a long time [1]. Based on anatomical location, urinary stones can be classified as kidney, ureteral, bladder, or urethral stones. Compositionally, they are categorized into calcium-based and non-calcium-based stones [2]. Calcium-containing stones, which appear radio-opaque, account for approximately 75% of all cases and are primarily composed of calcium oxalate and/or calcium phosphate. Less common types include carbapatite, uric acid, struvite (magnesium ammonium phosphate), and brushite stones [3]. The pathogenesis of UTSs remains incompletely understood and involves multiple contributing factors, including metabolic disorders, infections, dietary habits, fluid intake, and genetic predisposition [4,5,6]. Epidemiological evidence indicates that for every 5 kg/m^2^ increase in one’s body mass index (BMI), the relative risk of developing kidney stones increases by approximately 21%. Obesity, defined as a BMI ≥ 30 kg/m^2^, has been independently associated with a significantly elevated incidence of urolithiasis [7].

In 2000, Powell et al. [8] published the first study of obesity and kidney stones, which included 5942 patients with stones. Their urinalysis results showed that obese individuals exhibited an increased urinary excretion of calcium, oxalate, and uric acid, along with a lower urine pH, factors that promote lithogenesis. Subsequent studies further demonstrated that obesity alters the biochemical composition of urine, particularly its phosphate, oxalate, uric acid, and citrate levels [9,10]. Obesity also affects the kidney acid–base balance. Aciduria is observed in 54% of obese patients, a prevalence three times higher than that in individuals with a healthy BMI. Aciduria increases the risk of uric acid and oxalate stone formation [11,12]. Moreover, the high prevalence of diabetes among obese individuals may impair renal ammonium production and acid excretion, further lowering urinary pH and promoting stone formation [13].

The human microbiota is involved in the maintenance of health and susceptibility to mucosal site disease [14,15]. Recent improvements in detection methods have revealed increasing evidence for the presence of urogenital microbiota, challenging the long-standing belief that urine is sterile [16]. The microbiota has been shown to be associated with a variety of urinary system diseases, such as urinary tract infections, prostatitis, urinary incontinence, an overactive bladder, and urinary system tumors [17,18,19]. The role of microbiota in stone formation has attracted increasing research interest. We previously reported the microbiota to be associated with bladder cancer [20] and found that bladder cancer patients with three urinary diversion methods would have different urine microbiota [21]. Some researchers have studied the urinary microbiota of stone formers by 16S rRNA gene sequencing and observed distinct microbial differences between stone formers and healthy individuals [22,23,24]. In addition, recent research indicated that the makeup and organization of the urobiome play a significant role in both infectious and non-infectious stone formation, as specific microbes can serve as nucleation agents and enhancers in the development of lithiasis [25]. Dornbier et al. [26] used 16S rRNA gene sequencing and enhanced quantitative urine culture (EQUC) to find that several bacteria predominate in paired bladder urine and stone homogenates from the same patient. Liu et al. found that hypertensive kidney stone patients have unique urine microbiome characteristics [23]. Ursula et al. [16] found that microbiota may be associated with stone formation.

Currently, the mainstream methods for microbial detection primarily include metagenomic sequencing and 16S rRNA sequencing. However, metagenomic sequencing is limited by its high cost, while 16S rRNA sequencing targets only the 16S rRNA gene, typically allowing taxonomic resolution at the genus level, which restricts accurate and reliable identification at the species level. 2bRAD-M is a novel sequencing method for microbiota profiling that overcomes this limitation [27]. It utilizes type IIB restriction endonucleases to digest genomic DNA, generating uniform, isometric DNA fragments for high-resolution microbial analysis [27,28]. Amplification is used for sequencing and mapping Te fragments into species-specific 2bRAD markers for microbial characterization and quantification. 2bRAD-M can simultaneously detect three major classes of microbiota with a wider detection range. 2bRAD-M is based on thousands of unique tags on a genome-wide basis, combined with unique analysis algorithms, which can minimize false positives. 2bRAD-M has proven its ability to dissect low-biomass microbiota at the species level with high efficacy and at a low cost [27].

Sixteen stone patients were recruited and screened for this study, and urine was collected from their bladders. We performed 2bRAD-M to delineate the microbial community in their bladders with the aim of revealing the characteristics of the urinary microbiota in UTS patients with overweight UTS disease. To our knowledge, this is the first study to investigate differences in the composition of the urinary microbiota between overweight and healthy-weight UTS patients at a species-level resolution using 2bRAD-M.

## 2. Methods

### 2.1. Patient Recruitment

Twenty-five patients with unilateral stones from the Shanghai Tenth People’s Hospital were recruited. To minimize interference from external biological communities, strict exclusion criteria were applied: urinary tract infection, other urinary system diseases, history of surgeries unrelated to urinary tract stones, antibiotic treatment within the past 4 weeks, and catheterization within the past 4 weeks. Ultimately, a total of 16 patients met the above criteria through screening. All patients were diagnosed using CT scans and underwent lithotripsy. Stones removed during endoscopic surgery were analyzed for their chemical composition, and patient clinical information was collected.

### 2.2. Collection and Processing of Samples

**Figure 1:**
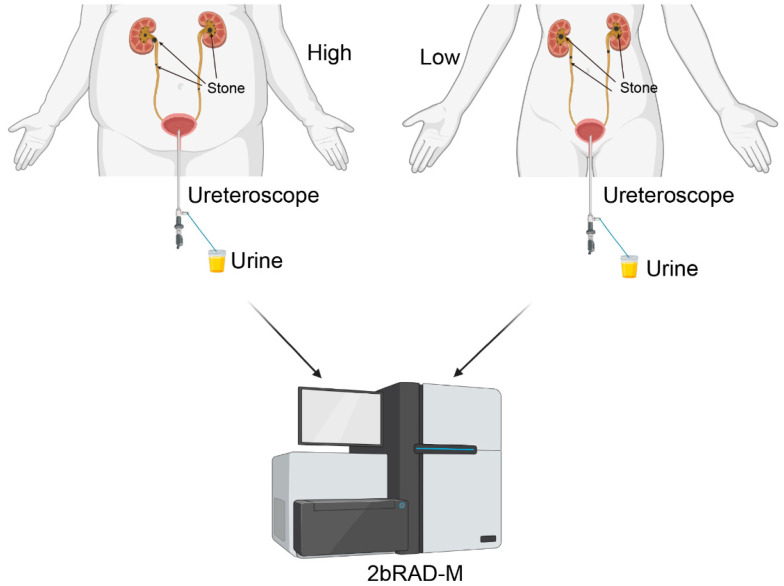
Schematic diagram of 2bRAD-M. Urine samples were taken from the bladders of overweight and healthy-weight UTS patients for 2bRAD-M testing. High: overweight UTS group; Low: healthy-weight UTS group.

The ethics review committee of the Tenth People’s Hospital approved the urine collection. Patients signed informed consent forms before sample collection. Urine samples were obtained from bladder urine before surgery, following the following procedure: Patients were placed in the lithotomy position, anesthetized, and disinfected. A ureteroscope was inserted into the urethra, and 15–20 mL of urine was collected using a sterile container before proceeding with lithotripsy. The urine collection workflow is shown in Figure 1. Samples were stored at below −80 °C within 30 min of collection, and the entire process was conducted under sterile conditions.

### 2.3. Dietary Data Collection and Analysis

All dietary data were collected during the first week of the 1st, 4th, 7th, and 10th postoperative months using the mobile application “Boohee” (https://www.boohee.com/appintros, access date: 13 February 2023). Participants were instructed to photograph their daily meals via the app, which provided automated nutritional assessments, including protein intake, fiber intake, water consumption, and total caloric intake. To minimize temporal bias due to seasonal variations in food availability and structural bias from dietary habit modifications, participants were advised to maintain their preoperative and postoperative dietary practices throughout the one-year study period. The potential renal acid load (PRAL) of their foods was calculated based on the method established by Remer and Manz [29]. For certain specialty foods not covered in their framework, PRAL values were derived from the Basica food database (www.basica.com/en/Alkaline-diet/Food-table, access date: 13 February 2023; Supplemental Material S2).

### 2.4. DNA Extraction, Library Preparation, and Sequencing

Genomic DNA was extracted using a TIANamp Micro DNA Kit (Tiangen, Beijing, China). The 2bRAD library preparation followed established protocols [16,19]. Initially, genomic DNA was digested with 4 U of BcgI enzyme (NEB, Ipswich, MA, USA) at 37 °C for 3 h. Subsequently, a ligation reaction was carried out at 4 °C for 16 h in a 20 μL reaction volume containing 10 μL of digested DNA, 0.2 μM of specific adapters (Ada1 and Ada2), 1 mM ATP (NEB), 1 × T4 DNA Ligase Buffer, and 800 U of T4 DNA Ligase (NEB). A thermal inactivation of BcgI was then performed at 65 °C for 20 min. The ligated DNA (7 μL) underwent PCR amplification in a 40 μL reaction mixture with each primer at 0.1 μM (primer 1 and primer 2 for Illumina), 0.3 mM dNTPs, 1 × Phusion HF Buffer, and 0.4 U of Phusion High-Fidelity DNA Polymerase (NEB). PCR cycles included denaturation at 98 °C for 5 s, annealing at 60 °C for 20 s, and extension at 72 °C for 10 s, with a final extension at 72 °C for 10 min. The Te library products were purified using the QIAquick PCR Purification Kit (Qiagen, Hilden, Germany) and sequenced on an Illumina HiSeq X Ten platform. Library construction and sequencing were performed by Qingdao OE BioTech Co., Ltd., Qingdao, China. The adapter and primer sequences are detailed in Supplementary Material S1: Table S1.

### 2.5. Sequencing Processing and Quantitative Analysis

Raw reads were filtered to extract enzyme reads based on the BcgI restriction enzyme recognition site. From these enzyme reads, clean reads were selected by applying the following criteria: (1) removing reads with more than 8% unknown bases, and (2) excluding reads containing over 20% low-quality bases (Q-value ≤ 20). The taxonomic profiling was performed using the 2bRAD-M computational pipeline (https://github.com/shihuang047/2bRAD-M, access date: 22 March 2024), which relies on a unique 2bRAD tag database (2b-Tag-DB) containing species-specific BcgI-derived tags identified from 173,165 microbial genomes (including bacteria, fungi, and archaea). Initially, clean reads were aligned against the prebuilt 2b-Tag-DB to identify microbial taxa present in each sample. A G score was computed for each identified species within a sample to control false-positive discoveries, calculated as $\sqrt{S_{i}\times t_{i}}$, where *S_i_* represents the number of reads assigned to all 2bRAD tags of species *i* within a sample, and *t_i_* represents the total number of all 2bRAD tags of species *i* sequenced within that sample. The G score, set with a threshold of 10 for false-positive species discovery, proved more sensitive than Bracken’s relative abundance estimation [16]. Candidate taxa were screened using this minimum G score criterion. To enhance quantitative accuracy, a secondary 2b-Tag-DB was developed specifically for these candidates, containing more tailored 2bRAD tags than the default database. The reads were then realigned against this sample-specific 2b-Tag-DB to determine the relative abundance of the candidate taxa. Subsequently, species’ relative abundance was computed as $\frac{S_{i}∕T_{i}}{\sum_{i=1}^{n}S_{i}∕T_{i}}$, where *S_i_* denotes reads assigned to all 2bRAD tags of species *i* within a sample, and *T_i_* represents the total number of 2bRAD tags for species *i*. Finally, a taxonomic abundance profile was generated [27,28].

### 2.6. Analysis of Microbial Diversity and Identification of Differential Taxa

Based on taxonomic abundance profiles, alpha diversity metrics such as the Shannon index and Simpson index were computed using the “vegan” package and visualized via boxplots [20]. Beta diversity was assessed using the same package and visualized through principal coordinate analysis (PCoA). A Venn diagram illustrated unique and shared species between the stone and non-stone sides. Linear discriminant analysis effect size (LEfSe) identified taxa differentially represented between the high and low sides, with a threshold set at a logarithmic LDA score of 2.0 [21]. To distinguish between the stone and non-stone sides, a random forest model was trained with tenfold cross-validation and 10 repetitions using the “randomForest” package, generating a cross-validation error curve [22]. The cutoff point was determined as the minimum cross-validation error plus its standard deviation (SD). All biomarker sets with errors below this cutoff were listed, with the optimal set defined by the fewest number of species.

### 2.7. Statistical Analysis

The clinical data were analyzed using SPSS (version 26.0) and R software (version 4.1.1). Categorical variables were presented as percentages, while continuous variables were expressed as median and interquartile range. Pairwise Wilcoxon rank-sum tests were applied to compare the alpha diversity and differences between microbial community groups. Beta diversity was assessed between the groups using a permutational multivariate analysis of variance (PERMANOVA). Spearman’s rank correlation was used to assess associations among microbial species based on their relative abundances. A *p*-value of <0.05 was considered statistically significant.

## 3. Results

### 3.1. Clinical Characteristics of Selected Patients

This study included 16 patients with unilateral urinary tract stones (UTSs), comprising 8 overweight patients and 8 healthy-weight patients. Table 1 lists the demographic and clinical characteristics of the selected patients. The median BMI was 27.02 (interquartile range (IQR): 26.67, 27.70) in the overweight group and 22.15 (IQR: 21.18, 22.93) in the healthy-weight group (*p* < 0.001). The healthy-weight group included five men and three women, with a median age of 60 years (IQR: 52–65). All eight patients in the overweight group were men, with a median age of 60.5 years (IQR: 46.75–68.5). There was no significant difference in age distribution between the two groups (*p* = 1.00). A total of seven patients were diagnosed with hypertension, including three (42.9%) in the healthy-weight group and four (57.1%) in the overweight group (*p* = 1.00). Similarly, diabetes was present in three patients: one (33.3%) in the healthy-weight group and two (66.7%) in the overweight group (*p* = 1.00). In terms of stone multiplicity, six patients (75%) in each group had a single stone, while two patients (25%) had multiple stones. We divided the location of the UTSs into left ureter, right ureter, left ureter + left kidney, and right ureter + right kidney, and there was no statistical difference between the two groups. In addition, the average maximum diameter of the UTSs in the healthy-weight group was 10 mm, while in the overweight group, it was also 10 mm. There was no significant statistical difference in the composition analysis of the UTSs between the two groups.

A comparative analysis between healthy-weight and overweight individuals revealed significant differences in several dietary and physiological parameters (Table 2). The median protein intake was 75.88 g (IQR: 64, 89) in the overweight group and 92.75 g (IQR: 79, 106) in the healthy-weight group (*p* = 0.0019). In contrast, the median fiber intake was significantly higher in the overweight group at 29.5 g (IQR: 25, 34), compared to 18 g (IQR: 14, 23) in the healthy-weight group (*p* < 0.001). The median potential renal acid load (PRAL) value was −3.24 (IQR: −7.5, 3.8) in the overweight group, significantly lower than 13.71 (IQR: 5.2, 18.7) in the healthy-weight group (*p* < 0.001). There were no significant differences in fermented food consumption between the two groups, with median values of 1.21 (IQR: 0, 2.7) and 0.96 (IQR: 0, 1.8) in the overweight and healthy-weight groups, respectively (*p* = 0.5156). The hydration level was also comparable between the groups, with a median of 2.62 (IQR: 1.3, 3.4) in the overweight group and 2.04 (IQR: 1.5, 3.2) in the healthy-weight group (*p* = 0.0727).

### 3.2. Biodiversity Analysis of Bacterial Communities in Urine

**Figure 2:**
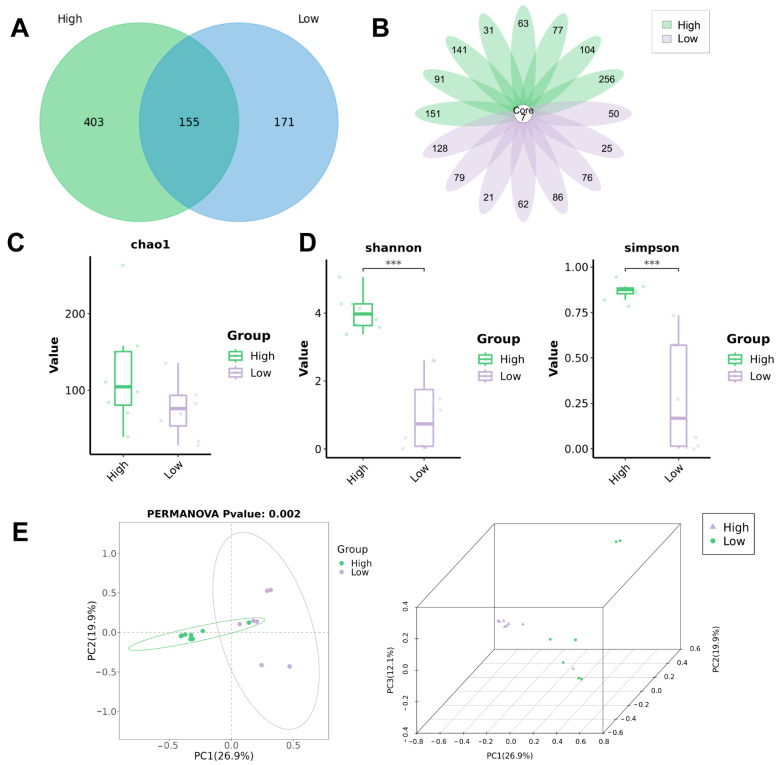
Biodiversity analysis of bacterial communities in urine. (**A**) Venn diagram of species of overweight and healthy-weight UTS groups; (**B**) flower plot of species of overweight and healthy-weight UTS groups. (**C**) Comparison of alpha diversity (Chao1) between the two groups. (**D**) Comparison of alpha diversity (Shannon index and Simpson index) between the two groups. (**E**) Comparison of beta diversity between the two groups based on PCoA. High: overweight UTS group; Low: healthy-weight UTS group. *** *p* < 0.001.

The clean reads were classified into 729 unique microbiota species based on their alignment against 2b-Tag-DB. As shown in the Venn diagram, there were a total of 155 identical species in the urine of the overweight and healthy-weight UST patients, accounting for 21.26% of the total species. There were 403 (72.22%) endemic species in the urine of the overweight UTS patients, and 171 (52.45%) endemic species in the urine of the healthy-weight UTS patients (Figure 2A). In addition, the results showed that all patients had seven identical species in their urine, namely *Bradyrhizobium sp002831585*, *Burkholderia sp018375725*, *Gordonia bronchialis*, *Ralstonia pickettii*, *Ralstonia sp000620465*, *Reyranella sp016780605*, and *Sphingomonas paucimobilis* (Figure 2B). For alpha diversity, Chao1 was calculated to evaluate the community richness of the urinary microbiota in the overweight and healthy-weight UTS patients, and the results showed no significant difference between the two groups (Figure 2C). The Shannon index and Simpson index were calculated to evaluate community diversity. The results showed significant differences in biodiversity between the two groups of microbial communities (Figure 2D). For beta diversity, differences between individuals and populations can be observed through PCoA, which shows significant differences in species composition between the two groups (Figure 2E).

### 3.3. Bacterial Community Composition in Urine of Overweight and Healthy-Weight UTS Patients

**Figure 3:**
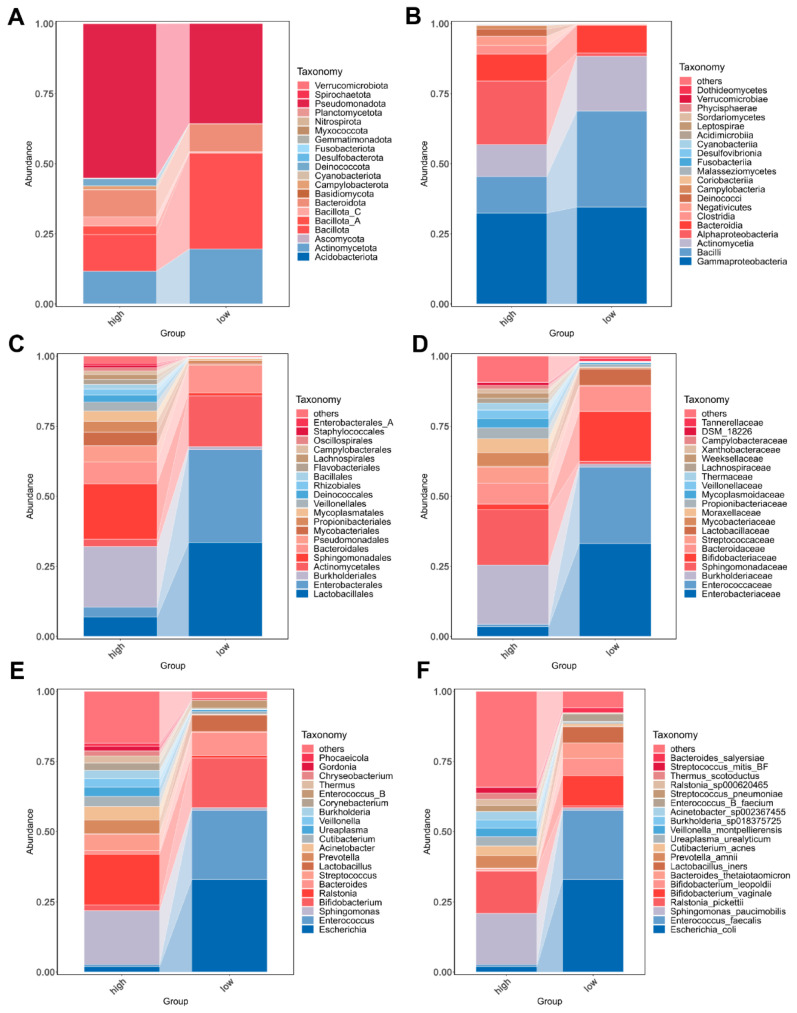
Microbial abundance and distribution in the two groups. The relative abundances of the top 20 most abundant microbial phyla (**A**), class (**B**), order (**C**), family (**D**), genera (**E**), and species (**F**) are represented in the barplot. High: overweight UTS group; Low: healthy-weight UTS group.

In order to explore the composition and dominant species in the urine microbiota of the patients in the grouping, we analyzed the community composition at the taxonomic levels of phylum, class, order, family, genus, and species. A total of 20 phyla were identified in the urine of the overweight UTS patients, while 14 phyla were identified in the urine of the healthy-weight UTS patients. Pseudomonadota was the most prevalent phylum in both groups, representing 55.06% in the overweight patients and 35.69% in the healthy-weight patients, followed by Bacillota (13.06% and 34.22%, respectively) and Actinomycetota (11.62% and 19.61%, respectively) (Figure 3A). At the class level, 32 classes were identified in the overweight patients, and 16 classes in the healthy-weight patients. Dominant classes in the overweight patients included Gammaproteobacteria (32.38%), Alphaproteobacteria (22.68%), and Bacilli (13.07%), while Gammaproteobacteria (34.57%), Bacilli (34.22%), and Actinomycetia (19.60%) were predominant in the healthy-weight patients (Figure 3B). At the same time, 63 and 37 orders were identified. Burkholderiales (21.68%), Sphingomonadales (19.68%), and Bacteroidales (7.78%) were the dominant orders in the urine of the overweight patients, and Lactobacillales (33.40%), Enterobacterales (33.15%), and Actinomycetales (18.16%) were the dominant orders in the urine of the healthy-weight patients (Figure 3C). At the family level, 116 families were identified in the overweight patients and 66 in the healthy-weight patients. The dominant families in the overweight patients included Burkholderiaceae (21.17%), Sphingomonadaceae (19.68%), and Bacteroidaceae (7.48%), whereas Enterobacteriaceae (33.15%), Enterococcaceae (27.25%), and Bifidobacteriaceae (17.78%) were the predominant families in the healthy-weight patients (Figure 3D). At the genus level, 263 and 133 genera were identified, respectively. *Sphingomonas* (19.21%), *Ralstonia* (18.05%), and *Streptococcus* (5.82%) were the dominant genera in the overweight patients; *Escherichia* (32.98%), *Enterococcus* (24.55%), and *Bifidobacterium* (17.78%) were the dominant genera in the healthy-weight patients (Figure 3E). Lastly, 558 and 326 species were identified. The dominant species of the overweight patients were *Sphingomonas paucimobilis* (18.29%), *Ralstonia pickettii* (15.09%), and *Prevotella amnii* (4.32%), and the dominant species of the healthy-weight patients were *Escherichia coli* (32.98%), *Enterococcus faecalis* (24.55%), and *Bifidobacterium vaginale* (10.70%). Supplementary Material S1 Figure S1 shows the top 30 different microbiota at the taxonomic levels of phylum, class, order, family, genus, and species in each overweight or healthy-weight patient with UTS. Figure S2 presents the top 10 most significantly different microbiota between the overweight and healthy-weight groups.

### 3.4. Different Abundance of Urinary Microbial Taxa in Grouped Patients

**Figure 4:**
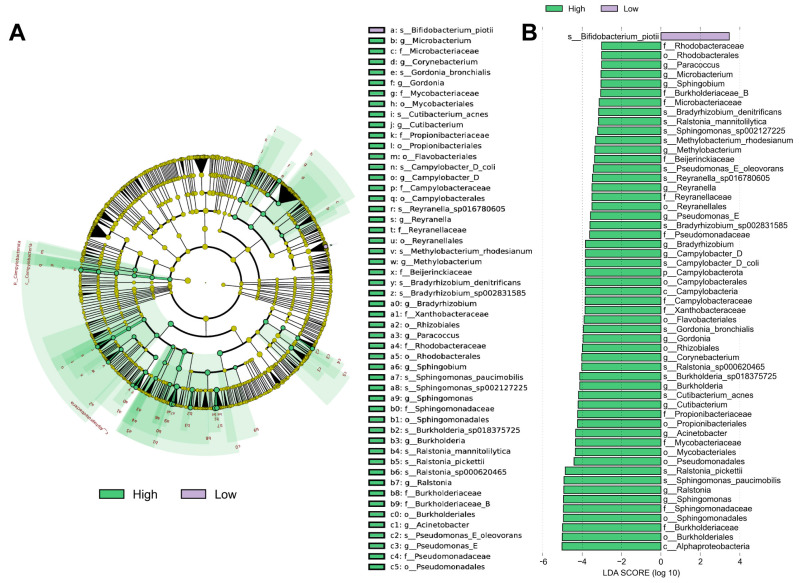
Different abundances of urinary microbial taxa in grouped patients. (**A**) The cladogram represents the taxonomic hierarchical structure of the identified 55 discriminative biomarkers using LEfSe. (**B**) The histogram of LDA scores showed 55 biomarkers with significant differences between the two groups. The LDA score represents the influencing degree of the biomarkers. High: overweight UTS group; Low: healthy-weight UTS group.

LEfSe was used to identify different abundant microbiota in both groups. We used LEfSe to identify 55 discriminant features (LDA score ≥ 2.0) with significantly different relative abundances in the overweight and healthy-weight groups (Figure 4A,B). The results showed that the species *Bilfidobacterium piotii* was abundant in the urine of healthy-weight UTS patients. The species enriched in the urine of overweight UTS patients were *Alphaproteobacteria*, *Burkholderiales*, *Burkholderiaceae*, *Sphingomonadales*, and *Sphingomonadaceae*. The microbiota that distinguished these two groups at the species level were mainly *Sphingomonas paucimobilis*, *Ralstonia pickettii*, *Cutibacterium acnes*, *Burkholderia sp018375725*, *Ralstonia sp000620465*, and *Bifidobacterium piotii* in the overweight and healthy-weight groups.

### 3.5. Correlation Between Microbiota

**Figure 5:**
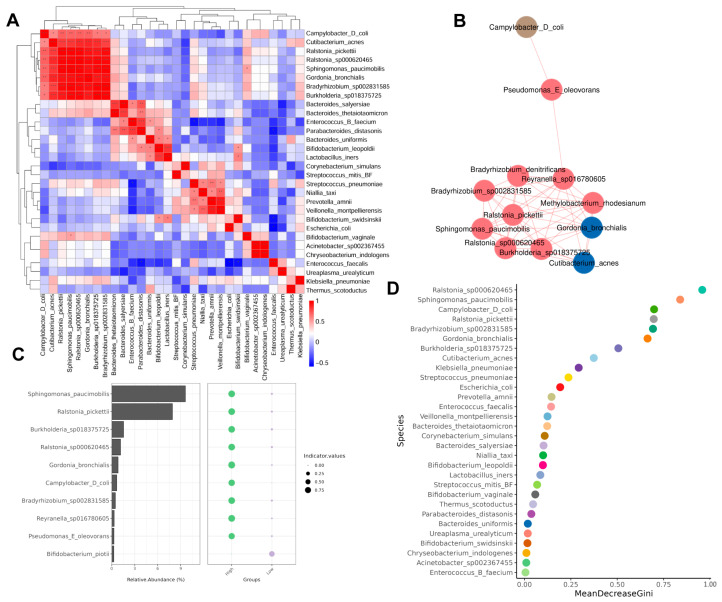
Correlation between microbiota. (**A**) Association heatmap between the top 30 rich communities of the two groups. (**B**) Association network map of the species with the highest correlation between the two groups. (**C**) Indicator analysis shows which species in the two groups have a greater impact on the growth environment. (**D**) Species importance point map of the top 30 abundance in the two groups. High: overweight UTS group; Low: healthy-weight UTS group. * *p* < 0.5, ** *p* < 0.01, *** *p* < 0.001.

A Spearman correlation analysis was performed to assess the association between the top 30 richest communities. The results showed that significant positive correlations were observed in different microbiota (Figure 5A), such as *Cutibacterium acnes* and *Ralstonia pickettii*, *Ralstonia sp000620465*, *Sphingomonas paucimobilis*, *Gordonia bronchialis*, *Bradyrhizobium sp002831585*, and *Burkholderia sp018375725*.

The species correlation network diagram mainly reflects the species correlation at each taxonomic level. We used the Spearman correlation coefficient calculation based on the relative abundance of species samples and constructed a species interaction network. We found that the species *Campylobacter D coli*, *Pseudomonas E oleovorans*, *Bradyrhizobium denitrificans*, *Bradyrhizobium sp002831585*, *Ralstonia pickettii*, *Reyranella sp016780605*, *Methylobacterium rhodesianum*, *Sphingomonas paucimobilis*, *Gordonia bronchialis*, and *Cutibacterium acnes*, as well as other species, are closely connected, indicating that there is a significant correlation between the others (Figure 5B).

In order to explore which microbial genera or species can have a greater impact on the growth environment within a certain area, we conducted an indicator analysis of the species. The results showed that *Sphingomonas paucimobilis* and *Ralstonia pickettii* had the highest urine abundance in the overweight group and had a greater impact on the growth environment, while *Bifidobacterium piotii*, although in lower abundance, could have a greater impact on the urine environment in the healthy-weight group (Figure 5C).

Species importance point maps can effectively and accurately classify microbial community samples and identify key components (species) that can distinguish differences between groups. We took the top 30 species in terms of their relative abundance to draw a species importance point map (Figure 5D). We found that the top five species with the highest importance were *Ralstonia sp000620465*, *Sphingomonas paucimobilis*, *Campylobacter D coli*, *Ralstonia pickettii*, and *Bradyrhizobium sp002831585*.

### 3.6. Functional Prediction Based on Microbiological Analysis Results

**Figure 6:**
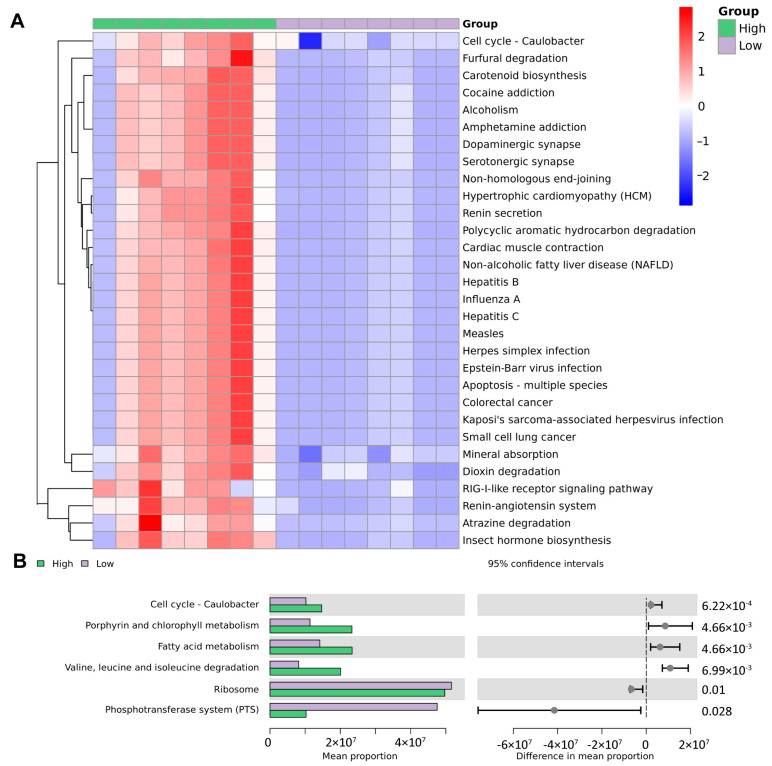
KEGG abundance analysis of microbial function. (**A**) KEGG difference result clustering heatmap. The top 30 with the smallest *p* value and the (**B**) KEGG difference result bar chart. The left bar chart shows the average abundance of the pathway in each group, and the right side shows the 95% confidence interval for the difference contrast between the groups and the corresponding significance *p* value. The top 6 difference items with the largest sum of the means of each group are shown. High: overweight UTS group; Low: healthy-weight UTS group.

To predict functional pathways from microbial community profiles, we performed a PICRUSt analysis (Phylogenetic Investigation of Communities by Reconstruction of Unobserved States) followed by KEGG pathway annotation (Kyoto Encyclopedia of Genes and Genomes). According to the clustering heatmap of the KEGG analysis, overweight and healthy-weight UTS patients have different urine microbial gene functions at different levels. As shown in Figure 6A and B, the urine of overweight UTS patients in the KEGG pathway analysis is significantly enriched. Microbial genes involve pathways such as those of the cell cycle of Caulobacter; porphyrin and chlorophyll metabolism; fatty acid metabolism; valine, leucine, and isoleucine degradation; ribosome; phosphotransferase system (PTS); polycyclic aromatic hydrocarbon degradation; renin secretion; renin–angiotensin system; and mineral absorption.

**Figure 7:**
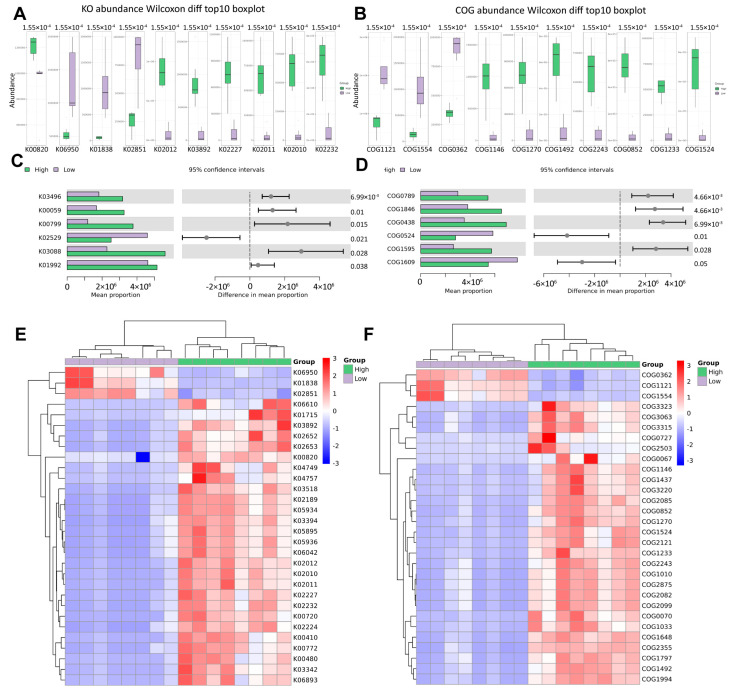
KO and COG abundance analysis of microbial function. (**A**,**B**) KO and COG abundance; Wilcoxon different top 10 boxplot. (**C**,**D**) KO and COG average abundance in each group. The right side is the 95% confidence interval for the difference between the groups and the corresponding significance *p* value. The top 6 difference items with the largest sum of the means of each group are shown. (**E**,**F**) KO and COG difference result cluster heatmap, showing the top 30 with the smallest *p* value. Left: KO; right: COG; High: overweight UTS group; Low: healthy-weight UTS group.

In addition, we also functionally annotated the genes by searching the KO gene cluster and Clusters of Orthologous Groups (COG) database. The following figure shows the first 10 differential KO and COGs (Figure 7A,B), as well as the average abundance of the first 6 differential KO and COGs in each group (Figure 7C,D). According to the cluster heat map of the KO and COG difference results (Figure 7E,F), we found that there are many differential urine microbial genes in overweight and healthy-weight UTS patients, which is consistent with our above analysis results.

## 4. Discussion

UTS is a multifactorial disease with a complex etiology involving both environmental and genetic factors [1]. To better understand its pathogenesis, a growing number of scientific and technological approaches have been applied to investigate the causes of stone formation [3,30,31,32]. Advances in 16S rRNA gene sequencing and EQUC have revealed complex and diverse microbial communities inhabiting the urinary tract of healthy individuals [33,34]. Since then, the urine microbiota has been associated with various urinary system diseases, including cancer and inflammation [35,36]. As one of the most prevalent conditions in urology, UTS has attracted increasing attention regarding the potential role of the urinary microbiota in its development. Recently, significant differences in the composition of the microbial community between stone patients and healthy individuals have been observed, which further suggests the potential importance of the urinary microbiota in stone formation [22,23]. Previous studies have demonstrated a close association between obesity and stone formation [37]. Obese individuals tend to present an increased urinary excretion of calcium, oxalate, and uric acid, along with a lower urine pH, all of which contribute to an increased risk of stone development [37,38]. However, the interplay between obesity, urolithiasis, and the microbiota remains insufficiently understood.

At present, most of the research on urine microbiota has used 16S rRNA sequencing to analyze the urinary microbiome. Most of the results cannot determine the taxa below the genus [39]. 2bRAD-M is a novel sequencing technology that maps microbial communities at the species level with high accuracy and at a low cost [27]. At present, 2bRAD-M has been used to detect many diseases related to the urinary microbiome, such as unilateral kidney stones, muscle-invasive bladder cancer, and non-muscle-invasive bladder cancer [40,41]. Here, we used 2bRAD-M technology for the first time to analyze the characteristics of the urine microbiota in overweight UTS patients, as well as to explore the differences and functions of urine microbial pathways in overweight UTS patients.

In the pathways found through the KEGG enrichment analysis of the 2bRAD-M results, we found that the urine of overweight UTS patients had differential pathways such as those of porphyrin and chlorophyll metabolism; fatty acid metabolism; valine, leucine, and isoleucine degradation; polycyclic aromatic hydrocarbon degradation; renin–angiotensin system; and mineral absorption.

Porphyrin and chlorophyll metabolism pathways were significantly enriched in the urine of overweight UTS patients [42]. Previous studies have shown that chlorophyll has the effect of delaying stone growth [43]. This finding suggests that microbiota in the urine of overweight UTS patients may affect stone formation and growth through this pathway. At the same time, the polycyclic aromatic hydrocarbon degradation pathway is also significantly enriched in overweight UTS patients. Multiple studies have confirmed that exposure to polycyclic aromatic hydrocarbons is associated with stone formation [42,44]. Our study revealed a significant enrichment of *Sphingomonas paucimobilis* in overweight patients. The research by Macchi et al. demonstrated that this bacterium regulates polycyclic aromatic hydrocarbon degradation (based on genomic/proteomic studies of strain 20006FA) [45]. Therefore, the presence of *Sphingomonas paucimobilis* in the urine of overweight patients may be directly associated with stone formation. Overweight patients may have an increased risk of UTS due to their reduced ability to metabolize polycyclic aromatic hydrocarbons, which affects urinary microbial changes. The mass spectrometry analysis by Aniello et al. [46] revealed significant changes in the amino acid profile in the urine of stone patients, especially revealing lower levels of α-aminobutyric acid, asparagine, ethanolamine, isoleucine, methionine, phenylalanine, serine, tryptophan, and valine. Valine, leucine, and isoleucine degradation further support the role of amino acid metabolism in stone formation, and the altered metabolism of amino acids may be caused by microbial changes. Obesity, especially the excessive distribution of visceral fat, leads to multiple alterations at the hormonal, inflammatory, and endothelial levels. These alterations may activate the renin–angiotensin system, thereby maintaining a hypertensive state [47]. In addition, studies have shown that the activation of the RAAS system can reduce calciuria and increase serum calcium, thereby reducing the risk of kidney stones [48]. This mechanism is also reflected in our 2bRAD-M results, suggesting that urinary microbial alterations in overweight UTS patients may be the result of the activation of the RAAS system.

It is noteworthy that the KEGG pathway is enriched in the fatty acid metabolism pathway, which is consistent with the existing research results that fatty acids and their metabolites play an important role in the pathogenesis of urinary stones. The study by Bikulčienė et al. [49] found that the amount of monounsaturated and polyunsaturated fatty acid intake directly affects stone formation, in which the percentage of polyunsaturated fatty acids in adipose tissue basically reflects the human diet. Compared with healthy individuals, patients with kidney stones have significantly higher monounsaturated fatty acid levels and lower polyunsaturated fatty acid levels. Siener et al. [50] found that patients with calcium oxalate stones may benefit from long-term supplementation with n-3 fatty acids. The mechanism of this physiological effect may be related to changes in the fatty acid pattern of membrane phospholipids and changes in the activity of oxalate transporters, which lead to reduced cellular oxalate exchange and reduce the risk of calcium oxalate stones. Hypercalciuria is a well-known risk factor for stone formation, and its causes are also associated with abnormal fatty acid metabolism. Baggio et al. [51] found that defects in the fatty acid composition of phospholipids may be the main cause of a series of metabolic and clinical changes in patients with kidney stones, including changes in kidney, intestinal, and bone calcium metabolism. And fatty acids are also closely related to stone prevention. Omar et al. [52] found that Omega-3 fatty acids combined with empirical dietary counseling can significantly reduce urinary calcium and oxalate excretion and increase urinary citrate in stone formers with hypercalciuria. This suggests that adjusting dietary fatty acid intake may help prevent stone formation. Through biotransformation and synthesis, gut microbes can convert lipids into bioactive metabolites that influence host physiology, particularly immune regulation and metabolic homeostasis [53]. Hall’s animal studies [54] further found that increasing dietary long-chain polyunsaturated fatty acids altered serum fatty acid concentrations and reduced the risk of urolithiasis formation in cats. *Prevotella amnii* was significantly enriched in the urine of overweight UTS patients; as a member of the *Prevotella* genus, it is potentially involved in fatty acid metabolic pathways, consistent with previous findings linking *Prevotella* species to lipid metabolism regulation [55,56]. Therefore, we hypothesize that the increased risk of UTS in overweight individuals may be related to altered fatty acid metabolism, potentially driven in part by differences in the composition of the urinary microbiota. Certain microbial taxa enriched in overweight individuals may participate in fatty acid metabolic pathways and contribute to this dysregulation. These changes may promote increased urinary calcium excretion and shifts in urinary microbial structure, facilitating the accumulation of crystals in the urinary tract and ultimately contributing to UTS formation.

According to existing studies on the urinary microbiota, UTS in overweight patients is unlikely to be caused by one microorganism or one pathway change. Urine contains multiple microbial communities that constantly influence each other, and the pathways that interact with each other continue to have an impact. Microbiota may change the chemical composition or pH value of urine to affect the lithogenic pathway, promote the growth and aggregation of calcium oxide crystals by affecting the surface structure, and induce renal tubular epithelial cells to express pro-inflammatory proteins and stone matrix proteins, thereby exacerbating stone formation. Protective pathways during stone formation may have anti-inflammatory functions and inhibit pathogenic bacteria colonization, thereby maintaining the balance of the microbiome and facilitating the formation of anti-pathogenic environments. We believe that the lithogenic and inhibitory pathways affected by urinary microbiota are key steps in the stone formation process in overweight people.

However, our study still has some limitations. First, the small sample size limits the statistical power and generalizability of our findings. As such, the results should be interpreted as exploratory and hypothesis-generating, providing a foundation for future studies with larger and more diverse cohorts. Second, all participants in the overweight group were male, leading to a gender imbalance that may influence urinary microbiota composition, as sex hormones are known to modulate microbial communities. Third, although antibiotic use within four weeks prior to sample collection was an exclusion criterion, other potential confounders such as hydration status, dietary intake, and probiotic use were not fully controlled. Notably, hydration level may affect urine concentration and thereby influence microbial DNA yield and diversity. These factors warrant further investigation in well-controlled studies. At the same time, we will collect more clinical data to retrospectively analyze the association between obesity and stones. These factors warrant further investigation in well-controlled studies. Fourth, the results of our study are based on correlation analyses of clinical samples using 2bRAD-M sequencing. It should be noted that all observed microbial associations represent correlations, and causality cannot be inferred from the current study design. Further experimental validation is required to obtain definitive conclusions.

## 5. Conclusions

Taken together, our findings shed light on the changes in the microbiome of the urine of overweight UTS patients, which involve multiple pathways associated with stone formation. With further research, we can better understand how these changes affect stone formation and provide new strategies for future prevention and treatment.

## Figures and Tables

**Table 1:** Baseline. Demographic and clinical characteristics of the selected patients.

Variables	Group		*p* Value
	Healthy-Weight (*n* = 8)	Overweight (*n* = 8)	
BMI	22.15 (21.18, 22.93)	27.02 (26.67, 27.70)	<0.001 *
Age	60 (52, 65)	60.5 (46.75, 68.5)	1 *
Gender			0.200 ^†^
Female	3 (100)	0	
Male	5 (38.46)	8 (61.53)	
Hypertesion	3 (42.85)	4 (57.14)	1 ^†^
Diabetes	1 (33.33)	2 (66.67)	1 ^†^
Stone count			1 ^†^
Solitary	6 (50)	6 (50)	
Multiple	2 (50)	2 (50)	
Stone side			0.688 ^‡^
Left ureter	4 (57.14)	3 (42.86)	
Left ureter and kidney	1 (50)	1 (50)	
Right ureter	1 (33.33)	3 (66.67)	
Right ureter and kidney	2 (66.67)	1 (33.33)	
Maximum diameter of stones	10 (8, 14.75)	10 (7.25, 22.25)	0.958 *
Stone composition			0.642 ^‡^
CaOx	3 (60)	2 (40)	
CaOx + CaP	1 (100)	0	
Urine acid	1 (33.33)	2 (66.67)	
None?	3 (42.86)	4 (57.14)	
Stone medical history	5 (50)	5 (50)	1 ^‡^

^*^ Mann–Whitney U test. ^†^ Fisher test. ^‡^ Chi-square test.

**Table 2:** Potential renal acid load (PRAL).

Variables	Group		*p* Value
	Healthy-Weight (*n* = 8)	Overweight (*n* = 8)	
BMI	22.15 (21.18, 22.93)	27.02 (26.67, 27.70)	<0.001
Calorie intake	2850 (2350, 3230)	1991.25 (1650, 2220)	<0.001
Protein intake	92.75 (79, 106)	75.88 (64, 89)	0.0019
Fiber intake	18 (14, 23)	29.5 (25, 34)	<0.001
PRAL value	13.71 (5.2, 18.7)	−3.24 (−7.5, 3.8)	<0.001
Fermented food consumption	0.96 (0, 1.8)	1.21 (0, 2.7)	0.5156
Hydration level	2.04 (1.5, 3.2)	2.62 (1.3, 3.4)	0.0727

## Data Availability

The data that support the findings of this study are available from the corresponding author upon reasonable request.

## Supplementary Materials

The following supporting information can be downloaded at https://www.mdpi.com/article/10.3390/biomedicines13051197/s1. Figures S1 (Microbial abundance and distribution in every patient.) and S2 (Statistical analysis at phyla, class, order, family, genera, and species.) and Table S1 (The sequences of adaptors and primers used in 2bRAD-M (5’-3’)) can be found in Supplementary Material S1. The Basica food database can be found in Supplementary Material S2.
