# Supplementary material for: 2bRAD-M Reveals the Characteristics of Urinary Microbiota in Overweight Patients with Urinary Tract Stones
Source: Biomedicines. 2025 May 14;13(5):1197. doi: 10.3390/biomedicines13051197 (PMC12108875; doi:10.3390/biomedicines13051197)
Supplement: Supplementary file 1 [file biomedicines-13-01197-s001.zip › Supplementary Material S1.pdf]

Article

2bRAD-M Reveals the Characteristics of Urinary Microbiota in Overweight Patients with Urinary Tract Stones

Pengfei Wu [1,2,3,†], Jingcheng Zhang [2,3,†], Wentao Zhang [2,3], Fuhang Yang [2,3], Yang Yu [2,3], Yuke Zhang [2,3], Guangchun Wang [2,3], Haimin Zhang [2,3], Yunfei Xu [2,3,*] and Xudong Yao [1,2,3,*]

1 Department of Urology, Shanghai Tenth People's Hospital, Clinical Medical College of Nanjing Medical University, Shanghai 200072, China

2 Department of Urology, Shanghai Tenth People's Hospital, School of Medicine, Tongji University, Shanghai 200072, China

3 Urologic Cancer Institute, School of Medicine, Tongji University, Shanghai 200072, China

* Correspondence: xuyunfeibb@sina.com (Y.X.); yaoxudong1967@163.com (X.Y.)

† These authors contributed equally to this work.

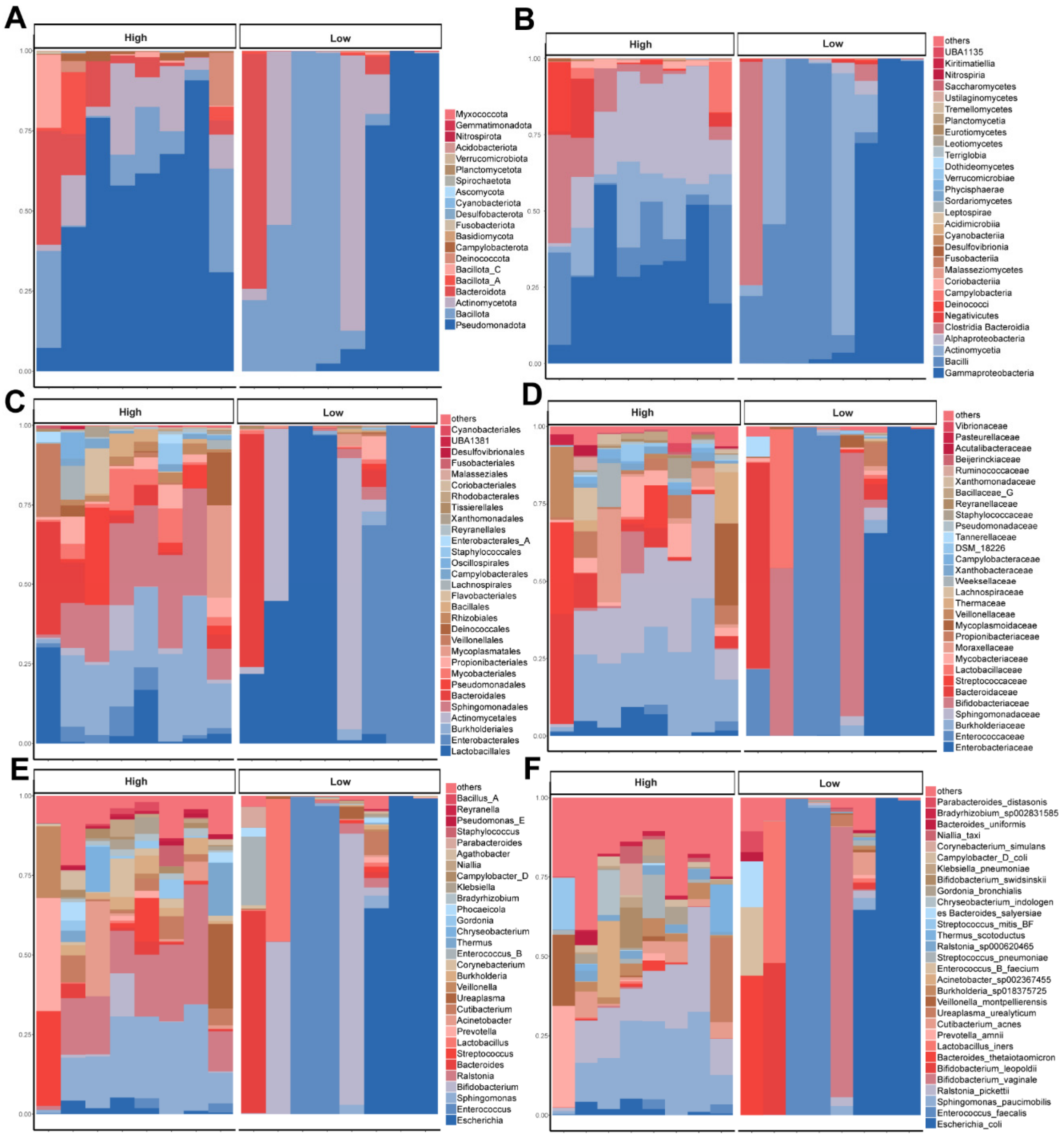

Figure S1: Microbial abundance and distribution in every patient. The relative abundance of the top 30 most abundant microbial phyla (A), class(B), order(C), family(D), genera (E), and species (F) is represented in the barplot. Show all of them when less than 30 microbes. High: overweight UTS group, Low: healthy-weight UTS group.

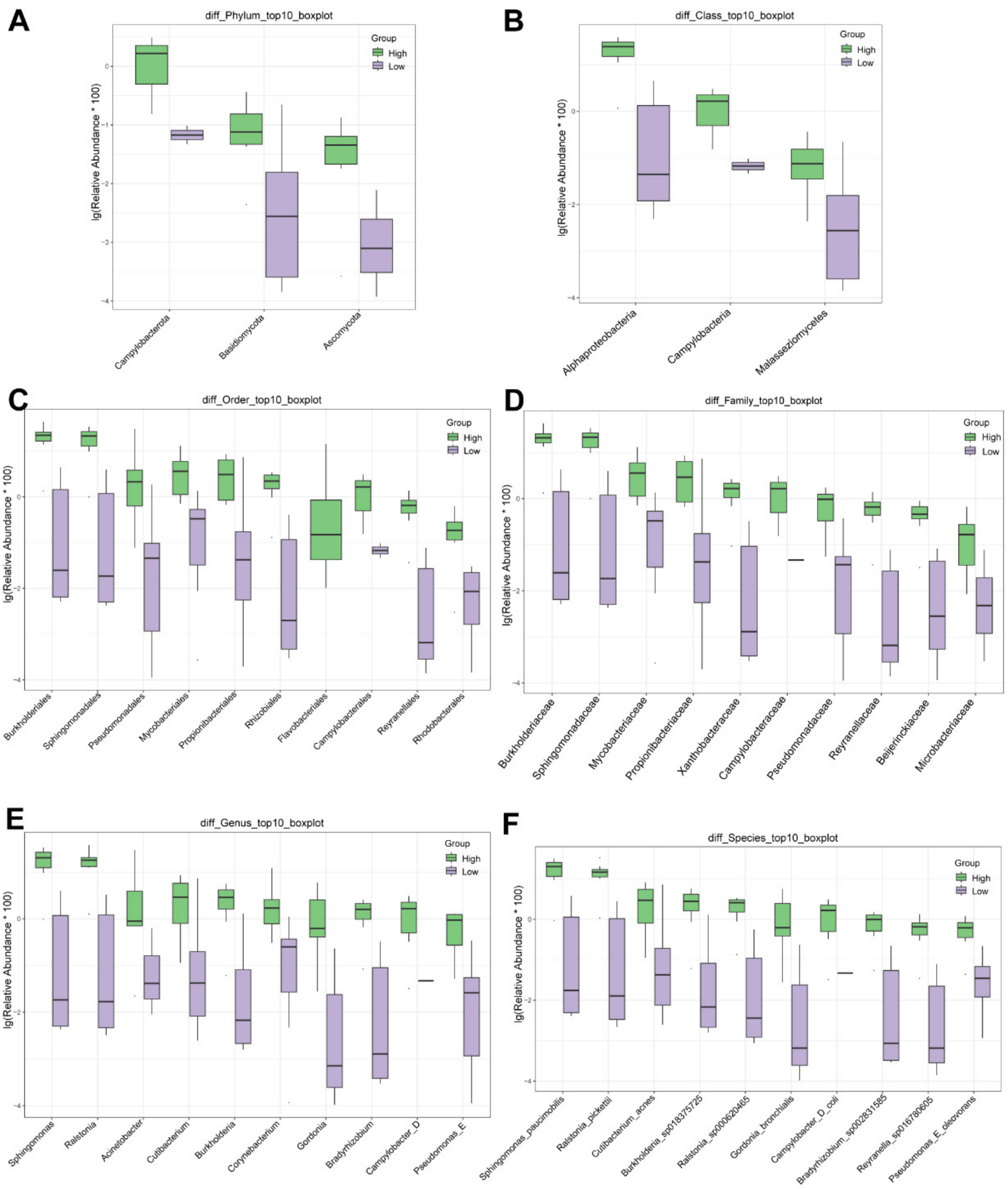

Figure S2: Statistical analysis at phyla (A), class(B), order(C), family(D), genera (E), and species (F), select the relative abundance boxplot analysis of the differential abundance microbial top10. Show all of them when less than 10 microbes. High: overweight UTS group, Low: healthy-weight UTS group.

Table S1. The sequences of adaptors and primers used in 2bRAD-M (5’-3’)

Adaptors	Sequence (5’-3’)
Adap-1 sense	ACACTCTTTCCCTACACGACGCTCTTCCGATCTNN
Adap antisense	AGATCGGAAGAGC
Adap-2 sense	GTGACTGGAGTTCAGACGTGTGCTCTTCCGATCTNN
Adap antisense	AGATCGGAAGAGC
Primer	
Primer1	ACACTCTTTCCCTACACGACGCT
Primer2	GTGACTGGAGTTCAGACGTGTGCT
5UDI Primer	AATGATACGGCGACCACCGAGATCTACACXXXXXXXXACACTCT TTCCCTACACGACGCTCTTCCGATCT
7UDI Primer	CAAGCAGAAGACGGCATACGAGATXXXXXXXXGTGACTGGAGT TCAGACGTGTGCTCTTCCGATCT
