# Supplementary material for: 2bRAD-M Reveals the Characteristics of Urinary Microbiota in Overweight Patients with Urinary Tract Stones
Source: Biomedicines. 2025 May 14;13(5):1197. doi: 10.3390/biomedicines13051197 (PMC12108875; doi:10.3390/biomedicines13051197)
Supplement: Supplementary file 1 [file biomedicines-13-01197-s001.zip › Supplementary Material S2.pdf]

Acid-Base Food Table

The table allows the assessment of dietary effects on acid-base balance. Foodstuffs with a negative value (milliequivalents per 100 g) exert a base (B) effect, foodstuffs with a positive value an acid (A) effect. Neutral foodstuffs are labelled with N.

Food	PRAL*	
	* Potential renal acid load (mEq/100g)	
**Beverages**		
Apple juice, unsweetened	B	-2,2
Beer, draft	B	-0,2
Beer, pale	A	0,9
Beer, stout	B	-0,1
Beetroot juice	B	-3,9
Carrot juice	B	-4,8
Coca-Cola	A	0,4
Cocoa, made with semi-skimmed milk	B	-0,4
Coffee, infusion, 5 minutes	B	-1,4
Espresso	B	-2,3
Fruit tea, infusion	B	-0,3
Grape juice	B	-1,0
Grape juice, unsweetened	B	-1,0
Green tea, infusion	B	-0,3
Herbal tea	B	-0,2
Lemon juice	B	-2,5
Mineral water (Apollinaris)	B	-1,8
Mineral water (Volvic)	B	-0,1
Orange juice, unsweetened	B	-2,9
Red wine	B	-2,4
Tea, Indian, infusion	B	-0,3
Tomato juice	B	-2,8
Vegetable juice (Tomato, beetroot, carrot)	B	-3,6
White wine, dry	B	-1,2
**Fats & Oil**		
Butter	A	0,6
Margarine	B	-0,5
Olive oil	N	0,0
Sunflower seed oil	N	0,0
**Nuts**		
Hazelnuts	B	-2,8
Peanuts, plain	S	8,3
Pistachio	S	8,5
Sweet almonds	S	4,3
Walnuts	S	6,8

Food	PRAL*	
	* Potential renal acid load (mEq/100g)	
**Fish & Seafood**		
Carp	A	7,9
Cod, fillets	A	7,1
Eal, smoked	A	11,0
Haddock	A	6,8
Halibut	A	7,8
Herring	A	7,0
Mussels	A	15,3
Prawn	A	15,5
Rose-fish	A	10,0
Salmon	A	9,4
Salted matie (herring)	A	8,0
Sardines in oil	A	13,5
Shrimps	A	7,6
Sole	A	7,4
Tiger Prawn	A	18,2
Trout, steamed	A	10,8
Zander	A	7,1
**Fruits**		
Apples	B	-2,2
Apricots	B	-4,8
Bananas	B	-5,5
Black currants	B	-6,5
Cherries	B	-3,6
Figs, dried	B	-18,1
Grapefruit	B	-3,5
Grapes	B	-3,9
Kiwi fruit	B	-4,1
Lemon	B	-2,6
Mango	B	-3,3
Orange	B	-2,7
Peache	B	-2,4
Pear	B	-2,9
Pineapple	B	-2,7
Raisins	B	-21,0
Strawberries	B	-2,2
Watermelon	B	-1,9

**Cereals & Flour**		
Amaranth	A	7,5
Barley (wholemeal)	A	5,0
Buckwheat (whole grain)	A	3,7
Corn (whole grain)	A	3,8
Cornflakes	A	6,0
Dried unripe spelt grains (wholemeal)	A	8,8
Dried unripe spelt grains (wholemeal)	A	8,8
Millet (whole grain)	A	8,6
Oat flakes	A	10,7
Rice, brown	A	12,5
Rice, white	A	4,6
Rice, white, boiled	A	1,7
Rye flour	A	4,4
Rye flour, wholemeal	A	5,9
Wheat flour, white	A	6,9
Wheat flour, wholemeal	A	8,2

**Pastries**		
Macaroni	A	6,1
Noodles	A	6,4
Spaetzle (German sort of pasta)	A	9,4
Spaghetti, white	A	6,5
Spaghetti, wholemeal	A	7,3

**Bread**		
Bread, rye flour	A	4,1
Bread, rye flour, mixed	A	4,0
Bread, wheat flour, mixed	A	3,8
Bread, wheat flour, whole meal	A	1,8
Bread, white wheat	A	3,7
Coarse wholemeal bread	A	5,3
Crispbread, rye	A	3,3
Pumpernickel	A	4,2
Rusk	A	5,9
Wholemeal bread	A	7,2

**Peas & Beans**		
Beans, green / French beans	B	-3,1
Lentils, green and brown, whole, dried	A	3,5
Peas	A	1,2

**Meat & Sausages**		
Beef, lean only	A	7,8
Cervelat sausage	A	8,9
Chasseur sausage	A	7,2
Chicken, meat only	A	8,7
Corned beef, canned	A	13,2
Duck	A	4,1
Duck, lean only	A	8,4
Frankfurters	A	6,7
Goose, lean only	A	13,0
Lamb, lean only	A	7,6
Liver (veal)	A	14,2
Liver sausage	A	10,6
Luncheon meat, canned	A	10,2
Ox liver	A	15,4
Pig's Liver	A	15,7
Pork sausage	A	7,0
Pork sausage (Wiener)	A	7,7
Pork, lean only	A	7,9
Rabbit, lean only	A	19,0
Rump steak, lean and fat	A	8,8
Salami	A	11,6
Slicing sausage containing ham	A	8,3
Turkey, meat only	A	9,9
Veal, fillet	A	9,0

**Milk, Dairy products & Eggs**		
Buttermilk	A	0,5
Camembert	A	14,6
Cheddar-type, reduced fat	A	26,4
Cottage cheese, plain	A	8,7
Cream, fresh, sour	A	1,2
Curd cheese	A	0,9
Edam Cheese full fat	A	19,4
Egg, chicken, whole	A	8,2
Egg, white	A	1,1
Egg, yolk	A	23,4
Emmental Cheese full fat	A	21,1
Fresh cheese (Quark)	A	11,1
Full-fat soft cheese	A	4,3
Gouda	A	18,6
Hard cheese	A	19,2
Kefir Cheese full fat	N	0,0
Milk, whole, evaporated	A	1,1
Milk, whole, pasteurised and sterilized	A	0,7
Parmesan	A	34,2
Processed cheese, plain	A	28,7
Rich creamy full fat cheese	A	13,2
Skimmed Milk	A	0,7
Whey	B	-1,6
Yogurt, whole milk, fruit	A	1,2
Yogurt, whole milk, plain	A	1,5

IHR TÄGLICHER AUSGLEICH. Basica

Sweats		
Chocolate, bitter	A	0,4
Chocolate, milk	A	2,4
Honey	B	-0,3
Ice cream, dairy, vanilla	A	0,6
Ice cream, fruit, mixed	B	-0,6
Madeira cake	A	3,7
Marmalade	B	-1,5
Nougat hazelnut cream	B	-1,4
Sugar, brown	B	-1,2
Sugar, white	N	0,0

Vegetables		
Asparagus	B	-0,4
Broccoli, green	B	-1,2
Brussel sprouts	B	-4,5
Carrots	B	-4,9
Cauliflower	B	-4,0
Celery	B	-5,2
Chicory	B	-2,0
Cucumber	B	-0,8
Eggplant	B	-3,4
Fennel	B	-7,9
Garlic	B	-1,7
Gherkin, pickeld	B	-1,6
Kale	B	-7,8
Kohlrabi	B	-5,5
Lamb's lettuce	B	-5,0
Leeks	B	-1,8
Lettuce	B	-2,5
Lettuce, iceberg	B	-1,6
Mushrooms, common	B	-1,4
Onions	B	-1,5
Peppers, Capsicum, green	B	-1,4
Potatoes	B	-4,0
Radish, red	B	-3,7
Ruccola	B	-7,5
Sauerkraut	B	-3,0
Soy beans	B	-3,4
Soy milk	B	-0,8
Spinach	B	-14,0
Tofu	B	-0,8
Tomato	B	-3,1
Zucchini	B	-4,6

Herbs & Vinegar		
Apple vinegar	B	-2,3
Basil	B	-7,3
Chives	B	-5,3
Parsley	B	-12,0
Wine vinegar, balamico	B	-1,6

© 2004 Klopfer-Nährmittel GmbH, D-85737 Ismaning. Modified according to Remer T, Manz F (1995) and Remer et al. (2003). All rights reserved. Reproduction, translation and commercial use including excerpts is prohibited without written consent of Klopfer-Nährmittel GmbH.
